# A Lightweight, Low-Frequency, Broadband Underwater Acoustic Transducer with Ternary Symmetric Excitation: Integrating KNN and Terfenol-D for Enhanced Performance

**DOI:** 10.3390/s26123645

**Published:** 2026-06-07

**Authors:** Xiongchao Ma, Zhenjun Liu, Shaobo Tang, Chenqi Shan, Qichao Li, Yiping Guo

**Affiliations:** 1 State Key Laboratory of Metal Matrix Composites, School of Materials Science and Engineering, Shanghai Jiao Tong University, Dong Chuan Road 800, Shanghai 200240, China; ma_xiongchao@163.com (X.M.); liqichao@sjtu.edu.cn (Q.L.); 2 Shanghai Marine Electronic Equipment Research Institute, Jindu Road 5200, Shanghai 201108, China; 13636393126@139.com (Z.L.); 13817918707@163.com (S.T.); s2338757836@foxmail.com (C.S.)

**Keywords:** underwater acoustic transducer, ternary symmetric excitation, KNN, Terfenol-D, lightweight, broadband and low frequency

## Abstract

Potassium sodium niobate (KNN) lead-free piezoelectric ceramics feature eco-friendliness and low density, coupled with superior high-frequency driving efficiency, albeit with inferior low-frequency performance. Conversely, Terfenol-D exhibits outstanding low-frequency driving capability but suffers from high density and poor high-frequency efficiency. This work proposes a ternary symmetric driving structure that integrates the complementary advantages of KNN and Terfenol-D, developing an underwater acoustic transducer with excellent lightweight design, low-frequency response, and broadband performance. The ternary symmetrically excited transducer maintains stable nodal planes across different operating frequencies and exhibits two distinct resonant frequencies. The vibration equation is analytically solved, and modal analysis is performed to clarify the evolution of the dual-resonance frequencies. A prototype transducer weighing 2.8 kg is fabricated and tested in an anechoic water tank. It delivers a maximum transmitting voltage response of 145 dB at 1.7 kHz with a broad operating bandwidth of 1–6 kHz. Compared with previously reported transducers, its weight is reduced by 26% to 93%. Benefiting from the double-ended radiation structure, the transducer yields a nearly omnidirectional radiation pattern. This ternary symmetrically excited transducer holds promising application prospects for underwater acoustic detection, communication, and navigation systems on unmanned underwater vehicle platforms.

## 1. Introduction

Underwater acoustic transducers are the core electromechanical conversion components in sonar systems, widely used in underwater detection, communication, navigation, and marine environmental monitoring. In recent years, the rapid development of unmanned underwater vehicles (UUVs) has placed increasingly stringent demands on underwater transducers, including lightweight construction, low-frequency resonance, broad operating bandwidth, high electroacoustic efficiency, and environmental friendliness. Traditional single-mechanism transducers—whether piezoelectric or magnetostrictive—fail to meet these requirements simultaneously due to inherent material limitations.

Hybrid excitation transducers that combine piezoelectric and magnetostrictive materials have garnered considerable attention by integrating the respective advantages of different functional materials. Since the 1990s, Butler and co-workers have proposed the first hybrid magnetostrictive/piezoelectric Tonpilz transducer based on lead zirconate titanate (PZT) and Terfenol-D, which achieves a lower resonant frequency and wider bandwidth than conventional transducers [1,2]. Subsequently, Downey and Mortensen developed a composite rod transducer by integrating lead magnesium niobate-lead titanate (PMN-PT) with Terfenol-D and established an analytical model for its mechanical impedance [3]. Chai et al. designed a concave barrel flextensional transducer with combined PZT and Terfenol-D actuation to address the narrow-bandwidth limitation of conventional transducers [4]. Teng et al. further achieved low-frequency and broadband operation in Tonpilz transducers through hybrid excitation of PZT and Terfenol-D [5]. Nevertheless, most existing hybrid transducers adopt a binary rod structure and face several critical bottlenecks: asymmetric mechanical impedance distribution, unstable nodal planes under variable-frequency excitation, and significant energy transmission loss. In addition, the high density of Terfenol-D and its matched magnetic bias assembly significantly increases the overall mass of the transducer, while high-frequency eddy-current loss degrades the electroacoustic efficiency at elevated frequencies [6,7]. Furthermore, the dominant use of lead-based PZT ceramics raises environmental concerns, conflicting with the development trend of green and sustainable marine devices.

To overcome these drawbacks, exploring novel material matching schemes and structural configurations is essential for high-performance underwater acoustic transducers. Lead-free potassium sodium niobate (KNN) piezoelectric ceramics have emerged as one of the most promising alternatives to PZT, owing to their eco-friendliness, low density (approximately 40% lower than PZT), and excellent high-frequency driving performance [8,9]. After years of material optimization—including texturing engineering [10,11], defect-dipole modulation [12,13], and doping modification—the piezoelectric coefficient, Curie temperature, and mechanical quality factor of KNN ceramics have been significantly improved, enabling them to compete with traditional lead-based piezoelectric ceramics [10,14]. Leveraging these advantages, KNN ceramics have been successfully applied in high-frequency ultrasonic transducers for biomedical imaging and nondestructive testing [15,16,17,18]. However, the relatively low electroacoustic efficiency of KNN at low frequencies limits its application in low-frequency underwater acoustic devices. In contrast, Terfenol-D, a giant magnetostrictive material, demonstrates outstanding low-frequency driving capability and large strain output, but its high density, high-frequency loss, and inductive impedance characteristics restrict its performance in the high-frequency range [19,20]. Clearly, KNN and Terfenol-D exhibit strong performance complementarity in density, frequency band, and driving mechanism, providing a feasible basis for developing high-performance hybrid transducers.

Fully exploiting the complementary advantages of KNN and Terfenol-D, this work proposes a ternary symmetrically excited underwater acoustic transducer with a K-T-K (KNN–Terfenol-D–KNN) configuration. The three-segment symmetric drive rod forms a fixed nodal plane at the central symmetry plane, thereby eliminating the impedance asymmetry and modal instability of traditional binary hybrid structures. Meanwhile, the low-density advantage of KNN reduces the total mass of the transducer, and the dual-resonance effect induced by ternary symmetric excitation effectively broadens the operating bandwidth. Terfenol-D dominates the low-frequency band, while KNN governs the high-frequency band, enabling balanced and high-efficiency electroacoustic conversion over a wide frequency range.

This paper systematically presents the structural design and working principle of the proposed ternary symmetric transducer. The dual-resonance broadband operating mechanism is revealed through lumped parameter modeling and vibration equation derivation, and finite element simulation is adopted to analyze the modal characteristics and vibration responses. A prototype is fabricated and tested in an anechoic water tank to validate its acoustic performance. Finally, the performance advantages of the proposed transducer are verified through quantitative comparison with state-of-the-art devices. This work provides a feasible technical solution for the design of lightweight, green, low-frequency, and broadband underwater acoustic transducers for UUV applications and next-generation green underwater acoustic systems.

## 2. Materials and Methods

### 2.1. Structural Design and Actuation Principle

**Figure 1:**
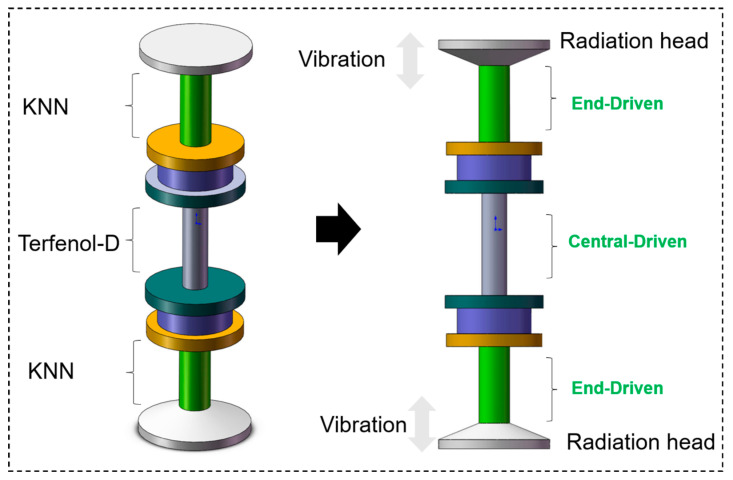
Ternary symmetrically excited transducer.

The proposed transducer features a ternary symmetrically excited structure consisting of three functional segments: KNN piezoelectric ceramics at both ends and Terfenol-D magnetostrictive material in the middle, forming a K-T-K (KNN–Terfenol-D–KNN) configuration. Detailed material parameters and structural dimensions are provided in the Supplementary Materials. As illustrated in Figure 1, the transducer adopts a central-end hybrid driving mode. Despite integrating two different functional materials, the overall structure maintains high symmetry with respect to the middle plane. As a result, a fixed zero-displacement nodal plane can be formed at the geometric center, independent of the operating frequency and vibration mode.

**Figure 2:**
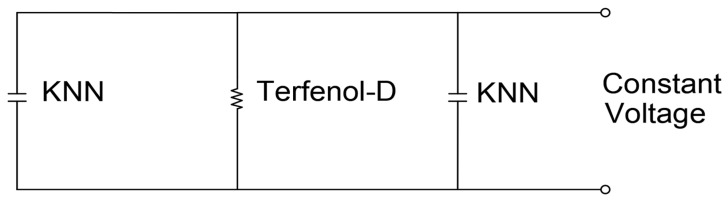
Parallel constant-voltage driving.

The mass blocks at the upper and lower ends act as dual acoustic radiation heads that vibrate symmetrically and radiate acoustic energy into water. This ternary symmetric structure based on KNN and Terfenol-D forms a double-ended radiation Tonpilz transducer, which can effectively mitigate energy leakage and enhance electroacoustic conversion efficiency. The hybrid excitation transducer has multiple signal input terminals, as both the rare-earth rod and the piezoelectric stack require electrical inputs. To the best of our knowledge, few specialized electrical connection schemes for such active materials have been reported in existing literature. In this work, we propose a parallel circuit configuration for the active materials, as shown in Figure 2, with a constant voltage source used for actuation. As demonstrated in the following analysis, this design allows the piezoelectric and magnetostrictive materials to operate efficiently within their respective frequency bands.

### 2.2. Dual-Resonance Low-Frequency Broadband Operating Mechanism

**Figure 3:**
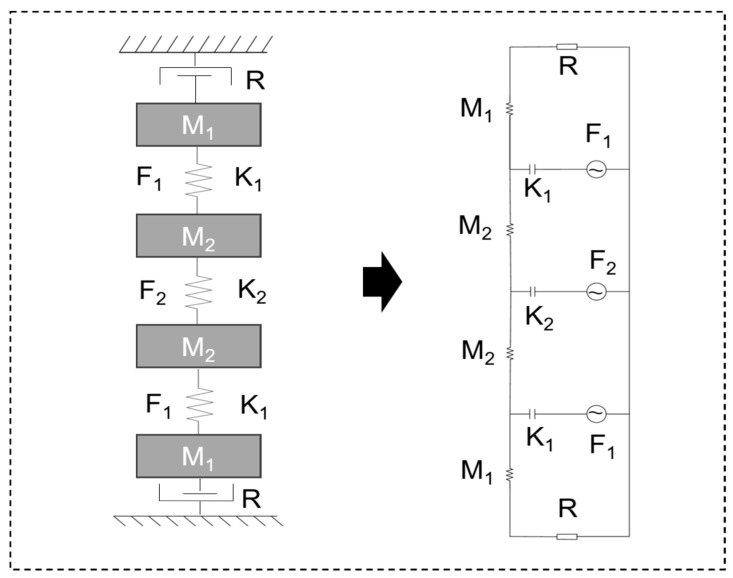
Lumped-parameter vibration model and symmetric mechanical impedance network.

The ternary symmetric structure can be simplified to a two-degree-of-freedom lumped-parameter vibration system, as shown in Figure 3. The dynamic model consists of mass blocks, equivalent springs, and damping elements.

Here, *m*_1_ denotes the mass of the radiation head, and *m*_2_ represents the lumped mass between the end-driven and central-driven rods. *k*_1_ and *k*_2_ represent the equivalent stiffness of the end-driven stack and central-driven stack, respectively, while *R* denotes the system damping. *F*_1_ and *F*_2_ are the forces generated by the end-driven and central-driven stacks. The vibration equation of the ternary symmetrically excited transducer is expressed as:

(1) $$\left[\begin{array}{cc} m_{1} & 0\\ 0 & m_{2} \end{array}\right]\left\{\begin{array}{c} {\ddot{x}}_{1}\\ {\ddot{x}}_{2} \end{array}\right\}+\left[\begin{array}{cc} R & 0\\ 0 & 0 \end{array}\right]\left\{\begin{array}{c} {\dot{x}}_{1}\\ {\dot{x}}_{2} \end{array}\right\}+\left[\begin{array}{cc} k_{1} & -k_{1}\\ -k_{1} & k_{1}-2k_{2} \end{array}\right]\left\{\begin{array}{c} x_{1}\\ x_{2} \end{array}\right\}=\left\{\begin{array}{c} F_{1}\\ F_{2} \end{array}\right\}$$

where x_1_ is the vibration displacement of m_1_, and x_2_ is that of m_2_. R represents the system damping. Solving this equation gives two resonant frequencies, as follows:

(2) $$\left\{\begin{array}{l} f_{i}=\frac{1}{2\pi }\sqrt{K_{ei}/M_{ei}},i=1,2\\ K_{e1}=\left[m_{1}k_{2}+m_{2}(k_{1}+k_{2})\right]-\sqrt{{\left[m_{1}k_{2}+m_{2}(k_{1}+k_{2})\right]}^{2}-4m_{1}m_{2}k_{1}k_{2}}\\ K_{e2}=\left[m_{1}k_{2}+m_{2}(k_{1}+k_{2})\right]+\sqrt{{\left[m_{1}k_{2}+m_{2}(k_{1}+k_{2})\right]}^{2}-4m_{1}m_{2}k_{1}k_{2}}\\ M_{e1}=M_{e2}=2m_{1}m_{2} \end{array}\right.$$

The transducer thus exhibits dual-resonance characteristics with two adjacent resonant peaks, which lay the foundation for broadband operation.

If $f_{0}=\frac{1}{2\pi }\sqrt{k_{1}/m_{1}}$ , under this condition, the following relation can be derived:

(3) $$f_{1}<f_{0}<f_{2}$$

The lower resonant frequency *f*_1_ is reduced relative to the intrinsic resonant frequency *f*_0_ of the original KNN actuator, whereas the higher resonant frequency *f*_2_ exceeds *f*_0_. Figure 3 presents the equivalent mechanical impedance circuit of the ternary symmetrically excited transducer. The diagram clearly shows a symmetric driving configuration that constructs an impedance network with inherent structural symmetry.

KNN lead-free piezoelectric ceramics are actuated by voltage under an electric field, and their strain vibration is in phase with the applied voltage. In contrast, Terfenol-D driving rods are driven by current under a magnetic field, and their strain vibration exhibits a 90° phase delay relative to the voltage. The three-element actuation system exhibits a 90° phase lead in the vibration of the central driving rod compared with that of the two end driving rods. Let the vibrations produced by the end-mounted KNN driving rods and the central rare-earth rod be defined as, respectively:

(4) $${\tilde{X}}_{1}(t)=X_{1}e^{jwt},\;{\tilde{X}}_{2}(t)=X_{2}e^{j(wt-\frac{\pi }{2})}$$

The combined vibration of the K-T-K ternary symmetric excitation transducer is thereby obtained as:

(5) $$\tilde{X}(t)={\tilde{X}}_{1}(t)+{\tilde{X}}_{2}(t)+{\tilde{X}}_{1}(t)\Rightarrow \tilde{X}(t)=(A-Bj)e^{jwt}$$

(6) $$\Rightarrow \left|\tilde{X}(t)\right|=\left|(A-Bj)e^{jwt}\right|=\sqrt{A^{2}+B^{2}}$$

(7) $$\Rightarrow \arg(\tilde{X}(t))=wt-a\tan(\frac{B}{A})$$

**Figure 4:**
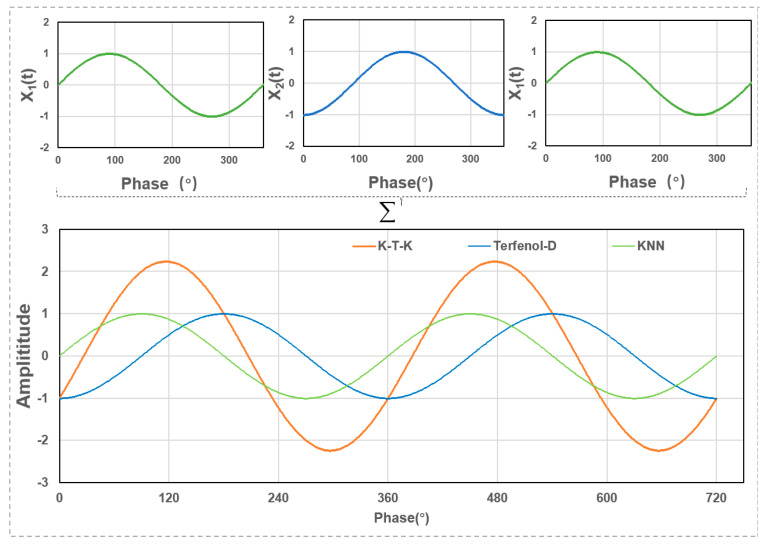
Displacement superposition of ternary symmetrical excitation.

Herein, *A* = 2*X*_1_, *B* = *X*_2_, and *w* denotes the angular vibration frequency. Accordingly, this excitation scheme allows efficient amplitude superposition of the three-segment materials, with only a limited phase shift relative to the driving signal, as shown in Equations (6) and (7). Set *X*_1_ = sin(*wt*) and *X*_2_ = −cos(*wt*). The superimposed displacement of the three-segment driven K-T-K transducer is thus derived, as shown in Figure 4.

**Figure 5:**
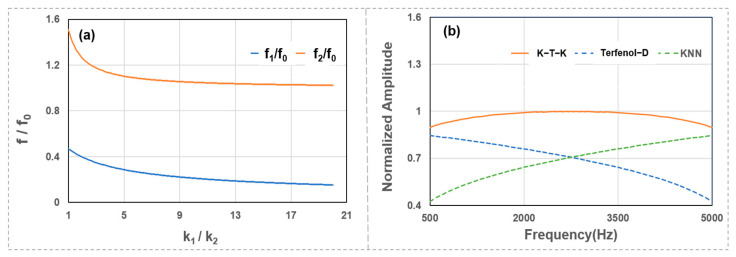
(**a**) Variation in the dual resonant frequencies as a function of the equivalent stiffness of the central driving rod. (**b**) Bandwidth performance of the K-T-K transducer.

Figure 5a depicts the variation in the normalized resonant frequencies *f*_1_/*f*_0_ and *f*_2_/*f*_0_ as a function of the mechanical compliance ratio k_1_/k_2_. In this mechanical impedance network, the ratio *k*_1_/*k*_2_ ranges from 1 to 20; *k*_1_ is kept constant while *k*_2_ is reduced, with *f*_0_ = 15 kHz and *R* = 0.5. It is evident from the curves that as *k*_1_/*k*_2_ increases, both normalized resonant frequencies *f*_1_/*f*_0_ and *f*_2_/*f*_0_ decline at first. This trend indicates that the resonant frequencies of the ternary symmetrically excited transducer drop significantly as *k*_2_ decreases. However, when *k*_1_/*k*_2_ > 2, only the lower resonant frequency *f*_1_ continues to decrease with further reduction in *k*_2_, while the higher resonant frequency *f*_2_ becomes nearly insensitive to changes in *k*_2_ (the numerical value of *f*_2_/*f*_0_ remains nearly constant at unity).

Owing to the inherently capacitive behavior of KNN, the impedance of the KNN actuator decreases with increasing frequency. At elevated frequencies, the majority of the current therefore flows through the KNN actuators. In contrast, the parallel-connected Terfenol-D actuators exhibit predominantly inductive behavior, resulting in higher impedance and, consequently, reduced current conduction. Consequently, at high frequencies, the system vibration is predominantly governed by the KNN actuators located at both ends. Conversely, under low-frequency excitation, the impedance of the Terfenol-D elements decreases, resulting in preferential current flow through these actuators and a corresponding reduction in the driving voltage across the KNN components. Due to the adoption of a constant-voltage drive, the respective powers generated by the KNN driving rod and the Terfenol-D driving rod for varying operating frequencies are as follows:

(8) $$\left\{\begin{array}{l} P_{KNN}=\left|\frac{2\pi U^{2}}{Z_{KNN}Q_{K}}\right|,Z_{KNN}=\frac{1}{jwC}\\ P_{Terfenol-D}=\left|\frac{2\pi U^{2}}{Z_{Terfenol-D}Q_{T}}\right|,Z_{Terfenol-D}=jwL \end{array}\right.\Rightarrow \left\{\begin{array}{l} P_{KNN}=\frac{2\pi wCU^{2}}{Q_{K}}\\ P_{Terfenol-D}=\frac{2\pi U^{2}}{wLQ_{T}} \end{array}\right.$$

In the formula, *P_KNN_* is the loaded power on KNN, *P_Terfenol-D_* on Terfenol-D, *Q_K_* and *Q_T_* the quality factor of the circuit, *C* the capacitance of the KNN driving rod, L the inductance of the copper coil with a rare-earth rod inserted in its center, and *U* the applied voltage. It can be observed that under such a parallel constant-voltage driving scheme for active materials, energy is mainly concentrated in KNN under high-frequency excitation, while it is mainly distributed in Terfenol-D under low-frequency excitation. Based on the static model (excluding the frequency characteristics of structural impedance), assuming that the vibration amplitude of KNN depends only on voltage, and that of Terfenol-D depends only on current, the vibration amplitude-frequency characteristics of K-T-K are obtained, as shown in Figure 5b.

In Figure 5b, Terfenol-D and KNN independently govern the low-frequency and high-frequency vibration regimes, respectively. By superimposing ternary symmetric actuation, an ultrawide operating bandwidth with a highly uniform (flat) frequency response is achieved. When the frequency characteristics of the KNN and Terfenol material structures are considered, due to the higher frequency constant of KNN and the lower frequency constant of Terfenol, this dual-frequency operation and modal coupling further broaden the transducer’s operating bandwidth.

### 2.3. Modal Analysis

Since the lumped-parameter model neglects the coupled vibration and distributed effects of masses and associated structures, it can only predict the number and approximate values of the natural frequencies of the system. This method is less accurate than finite element modal analysis and fails to obtain the displacement distribution of each vibrating component in practice. To verify the vibration characteristics and dual-resonance mechanism of the ternary symmetric structure, finite element modal analysis was performed using COMSOL Multiphysics 6.0. Three types of transducers with identical dimensions were established for comparison: PZT-based ternary actuation transducer, KNN-based ternary actuation transducer, K-T-K hybrid actuation transducer. The simulation results show that all three structures exhibit stable low-frequency f_1_ and high-frequency f_2_ modes with a fixed nodal plane at the central symmetry plane. The low-frequency mode corresponds to the coupled vibration of the radiation head and central mass, while the high-frequency mode corresponds to the independent vibration of the radiation head. By replacing the central material from KNN to Terfenol-D, the first-order resonant frequency is reduced, verifying that the K-T-K structure effectively lowers the fundamental resonance frequency.

**Figure 6:**
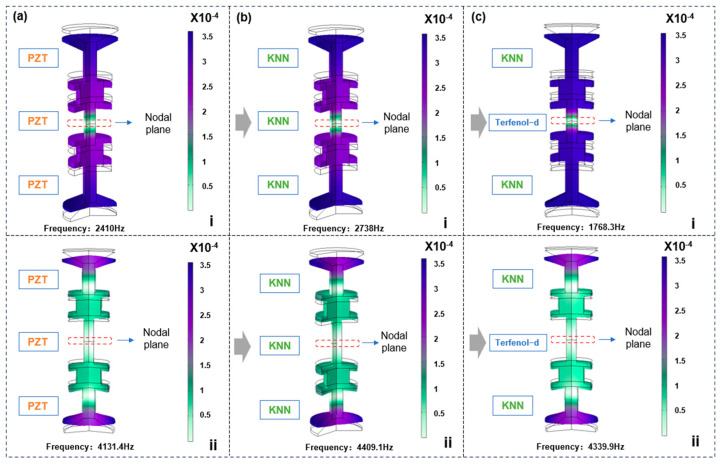
Vibration modes of the ternary symmetric actuation system: (**a**) PZT-based ternary actuation, (**b**) KNN-based ternary actuation, and (**c**) K-T-K ternary actuation configuration.

In Figure 6, all subpanels labeled “i” represent the low-frequency mode f_1_, whereas those labeled “ii” correspond to the resonant mode at f_2_. The results indicate that f_1_ originates from the coupled vibration of m_1_ and m_2_, with both the radiation head and the central mass exhibiting significant displacement amplitudes, as highlighted by the blue regions.

In contrast, f_2_ corresponds to a mode predominantly governed by the independent vibration of the radiation head, wherein only the radiation head exhibits a significant vibration amplitude, while the remaining regions undergo minimal motion. The vibration nodal plane of the transducer is located at the geometric mid-symmetry plane, as indicated by the dashed box. When the three-segment driving elements are replaced from PZT to KNN piezoelectric ceramics, the modal frequency increases from 2410 Hz to 2738 Hz, as shown in Figure 6(ai,bi). Furthermore, when the intermediate KNN actuator in Figure 6(bi) is replaced with Terfenol-D (Figure 6(ci)), the modal frequency decreases from 2734 Hz to 1768.3 Hz. Accordingly, the fundamental longitudinal resonant frequency of the transducer is reduced by 35.3% relative to the original PZT-based configuration. In contrast, the resonant frequency associated with the second degree of freedom exhibits only a marginal variation. This behavior is consistent with the previously discussed trend of f_1_, as it is primarily governed by the stiffness parameter k_2_. (It is worth noting that structural connections between the transducer housing and vibrator, and between Terfenol-D and its surrounding coils, are neglected in the model. These components are separated by sealing rings and air gaps and maintain a nearly decoupled state despite physical contact; such simplification is made for modeling convenience, and its influence on the frequency spectrum will be analyzed later. In addition, assembly-induced pre-stress on the driver and water loading effects on vibration modes are not included. Pre-stress has little effect on resonant frequency within a normal range. For the present piston-type longitudinal transducer with a small radiation area, water loading in non-deepwater environments barely changes the resonant frequency. The natural modes obtained in air remain present under water loading with similar frequency values).

### 2.4. Fabrication and Measurement

**Figure 7:**
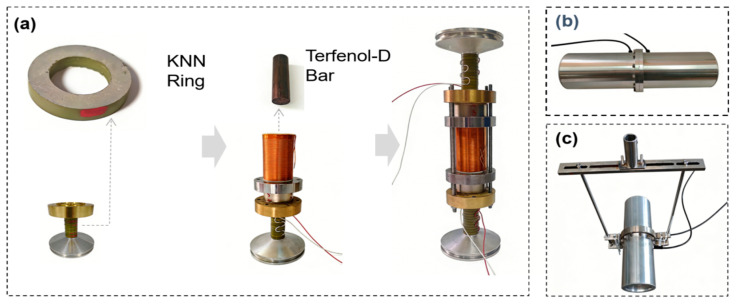
Fabrication and testing of the K-T-K transducer: (**a**) Fabrication of the K-T-K driving rod. (**b**) Assembly of the K-T-K transducer, and (**c**) Experimental test setup.

As depicted in Figure 7a, a lead-free KNN piezoelectric ceramic ring and a Terfenol-D rare-earth rod (As indicated by the dashed arrows in the figure) were employed to construct the piezoelectric vibrator and the hybrid rare-earth-piezoelectric actuation system, respectively. The Terfenol-D driving rod produces linear strain in response to variations in the applied magnetic field; accordingly, it is positioned within a solenoid coil wound with copper wire (1000 turns, inductance of 10 mH). The ternary symmetric vibrator is fixed mechanically using four external screws to ensure rigid coupling. The assembly is subsequently enclosed within a watertight protective housing with external feedthroughs, forming a double-ended, longitudinally radiating transducer, as illustrated in Figure 7b. The fabricated transducer prototype has an overall length of 280 mm, a diameter of 90 mm, and a total mass of 2.8 kg.

Figure 7c shows the K-T-K transducer mounted with a fixture for underwater testing. The central section of the transducer corresponds to a nodal plane across the entire broadband operating frequency range, where the displacement is minimal. Consequently, positioning the fixture at this location effectively reduces mechanical coupling between the transducer and the mounting structure. The instrumentation employed for the underwater tests included an oscilloscope (Keysight, DOSX3014T, Santa Rosa, CA, USA), signal generator (Keysight, 33500B, Santa Rosa, CA, USA), a power amplifier (Hangzhou Applied Acoustics Research Institute, Hangzhou, China), and an 8105 hydrophone (Hangzhou Applied Acoustics Research Institute, Hangzhou, China), with a sensitivity ranging from −205.7 dB to −205.2 dB over the frequency range of 20 Hz to 200 kHz.

## 3. Results and Discussion

**Figure 8:**
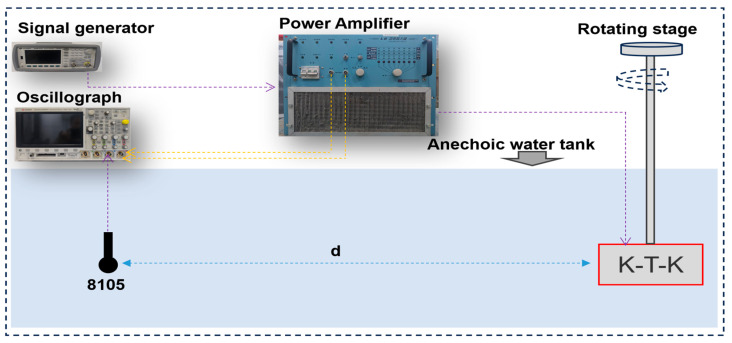
Experimental setup for the verification of transducer performance.

The measurement system shown in Figure 8 consists of a transmitting chain (a signal generator and a power amplifier) and a receiving chain (an 8105 hydrophone and an oscilloscope). A rotating stage was employed to characterize the spatial distribution of the acoustic field. Experiments were conducted in an anechoic water tank measuring 12 m × 8 m × 8 m. Both the transducer and the hydrophone were positioned at a depth of 4 m, with a separation distance of 6 m (The parameter d in the figure denotes this distance).

The K-T-K transducer operates via a hybrid voltage-current actuation mechanism. The transmitting voltage response (TVR) and transmitting current response (TIR) are defined as logarithmic measures of the sound pressure generated under unit-voltage and unit-current excitation, respectively, referenced to a standard sound pressure of 1 µPa. Using the underwater measurement system shown in Figure 8, TVR, TIR, and sound source level (SL) of the K-T-K prototype transducer were evaluated along its axial centerline. The corresponding calculation expressions are given as follows [21]:

(9) $$\left\{\begin{array}{l} TVR=20\log(\frac{V_{r}}{V}d)-M_{0}\\ TIR=20\log(\frac{V_{r}}{I}d)-M_{0} \end{array}\right.$$

In these equations, *V_r_* denotes the received signal measured by the 8105 hydrophone, d represents the separation distance between the transmitting transducer and the hydrophone, and *M*_0_ is the sensitivity of the 8105 hydrophone. The sound source level (SL) characterizes the acoustic output power of the transmitting transducer. It exhibits a linear relationship with both the TVR and TIR. The corresponding expression for SL is presented below [21]:

(10) $$\left\{\begin{array}{l} SL=TVR+20\log(U)\\ SL=TIR+20\log(I) \end{array}\right.$$

where U and I denote the root-mean-square voltages applied to the transducer and the current through the transducer, respectively. Based on measurements of the output voltage, current, and their phase difference *φ* of the power amplifier, the electroacoustic efficiency of the transducer under harmonic excitation can be determined by [22]:

(11) $$\eta =\frac{P_{acoustic}}{P_{electric}}=\frac{\frac{2\pi }{\rho_{w}c_{w}}\int_{0}^{\pi }p^{2}(\theta )\sin\theta d\theta }{UI\cos\varphi }$$

In which *η* denotes the electroacoustic efficiency of the transducer, *P_acoustic_* represents the acoustic power, and *P_electric_* corresponds to the input electric power. Therefore, it is necessary to monitor the loading voltage *U*, current *I*, and their phase difference *φ*. The two yellow dashed lines led from the power amplifier to the oscilloscope in Figure 8 serve as the monitoring channels for voltage and current signals, respectively. The term *p*(*θ*) describes the angular distribution of the sound pressure in the horizontal plane of the transducer under far-field conditions.

The K-T-K transducer incorporates three actuation channels, comprising two KNN channels and one Terfenol-D channel. Its performance was evaluated under three excitation configurations: three-channel K-T-K actuation, two-channel KNN actuation, and single-channel Terfenol-D actuation. The evaluated performance indicators include TVR, TIR, electroacoustic efficiency, and SL.

### 3.1. Directivity Pattern

To quantify the electroacoustic efficiency of the transducer, the sound field distribution p(θ) was measured, with the test focused on the vertical direction. The transducer features rotational symmetry in the horizontal plane; thus, the sound field in this cross-section is assumed to be uniformly distributed. In accordance with the measurement system illustrated in Figure 8, the transducer was mounted on a rotating platform. By adjusting the rotation of the platform, the sound pressure values in all directions of the transducer were acquired. Following the definition of the transducer directivity pattern, the position with the maximum sound pressure was set as the 0 dB reference. The resultant directivity pattern of the transducer is presented below.

**Figure 9:**
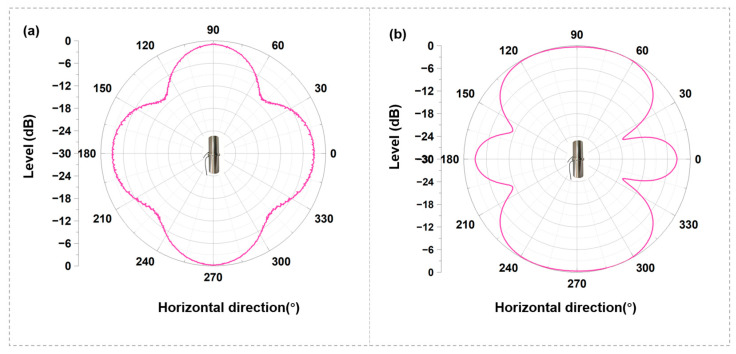
Horizontal directivity pattern: (**a**) 1.7 kHz directivity pattern, (**b**) 4.5 kHz directivity pattern.

Figure 9 presents the logarithmically normalized sound pressure distribution of the K-T-K transducer in the horizontal plane at f_1_ (1.7 kHz, Figure 9a) and f_2_ (4.3 kHz, Figure 9b). Compared with the typical applications of transducers driven by the binary combination of PZT and Terfenol-D, the hybrid-excited composite rod transducer proposed in this paper exhibits bidirectional and four-way radiation characteristics with four main lobes. At the first-order resonant mode (f_1_) of 1.7 kHz, as illustrated in Figure 9a, the four main lobes present a broad shape. Except for an approximately 10 dB attenuation at the azimuth of ±45°, the acoustic energy is uniformly distributed in most directions, achieving a wide coverage of acoustic radiation. As the operating frequency increases, the attenuation at ±45° becomes more significant. Nevertheless, the transducer maintains a −6 dB beamwidth of nearly 110° at both radiation ends, retaining a large effective radiation area.

Accordingly, this transducer does not require precise alignment with communication targets, making it well-suited for wide-area underwater acoustic communication. It is particularly applicable to underwater sensor networks, underwater formation networking, broadcast communication of buoy nodes, and random communication of unmanned underwater vehicles (UUVs). This design can effectively avoid communication interruptions caused by carrier attitude deviation and position drift. Meanwhile, the wide-range radiation performance mitigates signal distortion induced by underwater multipath effects, thereby ensuring stable communication links among multiple nodes over a large area.

In the field of underwater acoustic navigation, the omnidirectional radiation mode enables continuous transmission of reference navigation acoustic signals in all directions. It satisfies the requirements of omnidirectional positioning and route calibration for underwater submersibles, underwater robots, and underwater operating equipment, and breaks the azimuth limitation of directional beams. The proposed transducer can be deployed for regional navigation networking in extensive water areas and also serves as an underwater reference beacon, which greatly improves the coverage and operational flexibility of navigation systems.

### 3.2. Electroacoustic Performance

**Figure 10:**
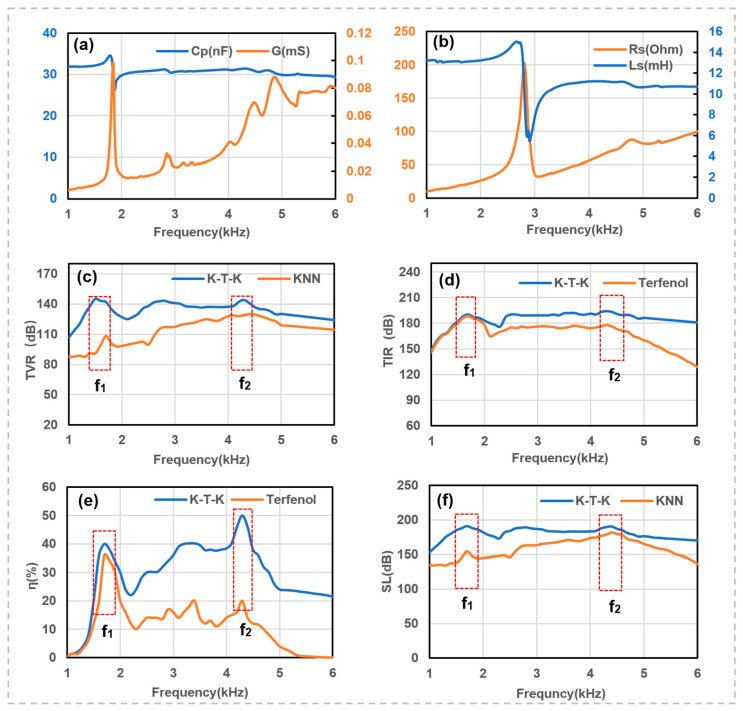
(**a**) The Cp-G curves of the KNN channels, (**b**) the Ls-Rs curves of the Terfenol-D channel, (**c**) transmitting voltage response (TVR), (**d**) transmitting current response (TIR), (**e**) electroacoustic efficiency, (**f**) sound source level (SL) polar pattern.

For the K-T-K transducer, we focus on the comparative analysis of electroacoustic characteristics among independent Terfenol-D driving, two-channel KNN driving, and three-port K-T-K hybrid driving schemes in the prototype. First, the capacitance-conductance characteristics of the parallel dual-channel KNN unit and the inductance-resistance characteristics of the Terfenol-D channel were measured underwater, as presented in Figure 10a,b. Distinct resonant peaks are observed near 1.8 kHz and 4.8 kHz for the parallel dual-channel KNN piezoelectric unit in Figure 10a, which is well consistent with the lumped parameter model and modal analysis presented earlier. The minor discrepancies originate from the difference in physical mechanisms: the theoretical model mainly focuses on the resonant frequency of mechanical vibration, while the measured results reflect the conductance resonance under electromechanical coupling.

In Figure 10b, an anti-resonance peak of resistance appears around 2.7 kHz. This characteristic indicates a potential trough in the subsequent electroacoustic response curves, since a lower current corresponds to weaker driving capability for magnetostrictive materials. The two characteristic frequencies f_1_ and f_2_ derived from the follow-up electroacoustic response curves show better agreement with the theoretical predictions. This is because the acquired signals are acoustic amplitude responses converted from mechanical energy, which are collected via the hydrophone.

As shown in Figure 10c, the K-T-K transducer possesses two resonant frequencies f_1_ and f_2_. The measured values of the prototype are f_1_ = 1.7 kHz, f_2_ = 4.3 kHz. The TVR curve of the ternary-driven K-T-K transducer is significantly flatter than that of the pure KNN transducer, with an overall increase exceeding 10 dB. Notably, in the low-frequency range, the TVR of the K-T-K configuration is nearly 30 dB higher than that of the pure KNN transducer. The TVR of the K-T-K transducer exceeds 140 dB at 1.7 kHz and remains approximately 140 dB over the frequency range of 2.5–4.4 kHz with fluctuations of less than 3 dB. Over the 1–6 kHz operating band, the TVR varies from 110 to 145 dB. Compared with conventional longitudinal vibration transducers, the proposed design exhibits a lower operating frequency limit and a broader, more uniform (flatter) frequency bandwidth [23,24]. Furthermore, the absence of bending modes is expected to confer enhanced structural robustness, enabling operation at greater underwater depths.

Figure 10d compares the current response of the K–T–K transducer with that of the pure Terfenol-D-driven configuration. The two curves exhibit similar trends, with substantial overlap in the low-frequency region, indicating that the K-T-K transducer’s low-frequency response is predominantly governed by Terfenol-D actuation. Furthermore, as the frequency increases, the TIR of the K-T-K transducer progressively exceeds that of the pure Terfenol-D configuration. In the high-frequency region, the TIR of the K-T-K transducer is approximately 5–10 dB higher than that of the Terfenol-D transducer.

Figure 10e presents the electroacoustic efficiency of the transducer, calculated using Equation (10). The K-T-K transducer achieves efficiencies exceeding 40% at the resonant frequencies f_1_ and f_2_. Compared with the pure rare-earth-driven configuration, the electroacoustic efficiency is markedly enhanced over the frequency range from f_1_ to f_2_. Within the frequency band of 1.5 kHz–5 kHz, the electroacoustic efficiency of the transducer ranges from 22% to 50%.

Figure 10f shows the SL of the transducer under applied voltages of 100–500 V and currents of 0.001–1.1 A. The results indicate that compared with the fully KNN-driven configuration, the K-T-K transducer exhibits a substantial enhancement in SL within the low-frequency region near f_1_, with a maximum increase of approximately 32 dB. Across the operating frequency range of 1–6 kHz, the SL varies between 155 and 192 dB.

### 3.3. Comparative Analysis with Literature Transducers

A comprehensive comparison was conducted between the fabricated transducer prototype and the transducers reported in refs. [2,4,25] in terms of dimensions, weight, electroacoustic performance, and other aspects, as summarized in Table 1.

Ref. [2] presents a classic longitudinally vibrating transducer with hybrid excitation composed of PZT and Terfenol-D. Although its transmitting voltage response (TVR) is higher in the frequency range of 1–6 kHz (124–152 dB for ref. [2] versus 109–145 dB in this work), the proposed transducer achieves a remarkable reduction in weight. Accordingly, it exhibits higher electroacoustic efficiency and TVR per unit mass. The weight of the developed transducer is approximately 1/14 of that in ref. [2] (40 kg for ref. [2] and 2.8 kg for this work). If 14 identical transducers proposed herein are used, the overall TVR will increase by 23 dB (20 × log (14) = 23), reaching 131–168 dB, which is evidently superior to that in ref. [2]. Although the beam pattern becomes narrower, the designed transducer features bidirectional and four-way radiation with four lobes, as illustrated in Figure 9. Consequently, it generates more acoustic energy under the excitation of unit mass and unit driving voltage.

In ref. [4], the low-order resonant frequencies are mainly generated by the flexural vibration of the radiating shell, while the high-order modes originate from the longitudinal vibration of the driving rod. By contrast, the low-order modes of our transducer are directly excited by the driving rod. The transducer in ref. [4] exhibits lower transmitting voltage response (TVR) and electroacoustic efficiency, with a TVR range of 126–134 dB. More critically, its low-frequency performance relying on flexural modes of the shell is not suitable for deep-water service. Furthermore, it also features larger dimensions and a heavier weight.

**Figure 11:**
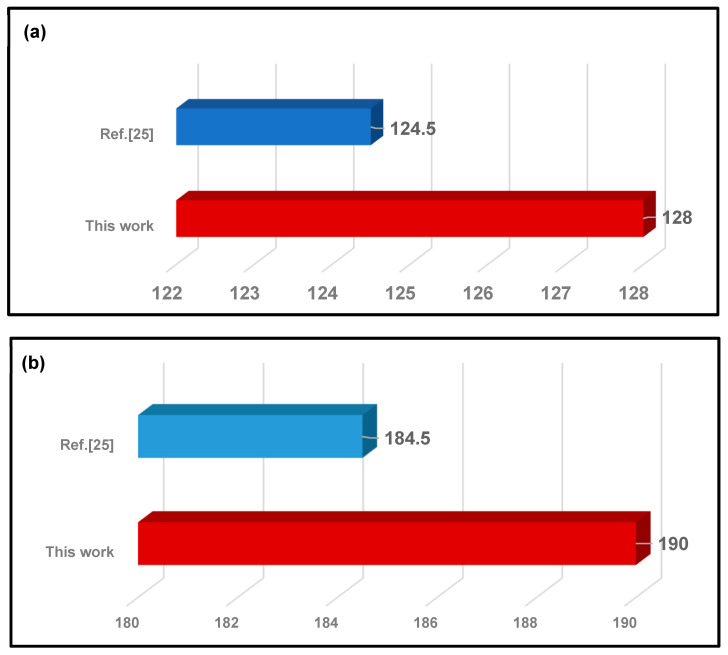
(**a**) Comparison of TVR under single-voltage driving (dB), (**b**) Comparison of TIR under independent current driving (dB).

Ref. [25] reports an ultra-compact rare-earth/piezoelectric hybrid transducer with a weight comparable to the present design (2.6 kg for the transducer in ref. [25] and 2.8 kg for this work). The full frequency response curve is not provided in the literature; only two resonant frequencies of 1.82 kHz and 3.76 kHz, as well as the maximum transmitting voltage response (TVR) under independent voltage excitation and independent current excitation, are presented. These two resonant frequencies are close to those of our transducer, thus enabling a valid comparison of their electroacoustic performances. The detailed comparison between the two transducers is given below (Figure 11):

It can be observed that the weight difference between the two transducers is only approximately 10%. Nevertheless, their transmitting voltage response and current response differ by 3.5 dB (Figure 11a), 5.5 dB (Figure 11b) under identical excitation conditions. This indicates that the acoustic energy output of the proposed transducer is nearly doubled per unit driving voltage or current.

In summary, the proposed transducer achieves substantial improvements in overall size, weight, electroacoustic efficiency and operating bandwidth compared with previous designs. In particular, a remarkable enhancement is realized in electroacoustic efficiency per unit mass. A CW pulse with a duty cycle of 1/4 was used for excitation in all tests. The THD was measured to be 1–2.3% across 1 kHz–10 kHz. Owing to the asymmetric S-E curve induced by dipole doping in KNN materials, the output signal of the KNN piezoelectric rod has around 10% offset and a noticeable DC component in the spectrum, which does not fundamentally hinder its practical use.

## 4. Conclusions

A novel ternary symmetrically excited underwater acoustic transducer with a K-T-K (KNN–Terfenol-D–KNN) configuration is proposed, fabricated, and tested in this work. By combining the complementary advantages of KNN lead-free piezoelectric ceramics and Terfenol-D giant magnetostrictive material, the transducer achieves lightweight, low-frequency, broadband, high-efficiency, and near-omnidirectional radiation performance simultaneously. Compared with conventional hybrid transducers, the proposed transducer overcomes the drawbacks of heavy mass, narrow bandwidth, high energy loss, and lead contamination. It provides a promising, eco-friendly, and high-performance solution for underwater acoustic detection, communication, and navigation systems, especially for lightweight, highly integrated unmanned underwater vehicle (UUV) platforms.

## Figures and Tables

**Table 1:** Comparison of the K-T-K ternary symmetric excitation transducer with literature-reported hybrid excitation transducers.

Parameters	Reference [2]	Reference [4]	Reference [25]	This Work
Dimension (mm)	Diameter: 214 Length: 406	Diameter: 88 Length: 316	Diameter: 54 Length: 235	Diameter: 90 Length: 280
Weight (kg)	40	3.8	2.6	2.8
Modal (kHz)	1.8, 3.5	1.3	1.82, 3.76	1.7, 4.3
Bandwidth (kHz)	1–6	1–4	1–4	1–6
Acoustic field distribution	Unilateral radiation	Unilateral radiation	Unilateral radiation	Four-terminal radiation
Maximum.TVR (dB)	152	134	124.5	145
Transducer type	Tonpilz	flextensional	Tonpilz	Tonpilz
Excitation method	Dual excitation of PZT and Terfenol-D	Dual excitation of PZT and Terfenol-D	Dual excitation of PZT and Terfenol-D	Ternary symmetric excitation using KNN and Terfenol-D

## Data Availability

Research data will be available upon reasonable request.

## Supplementary Materials

The following supporting information can be downloaded at: https://www.mdpi.com/article/10.3390/s26123645/s1, Figure S1: Ternary symmetrically excited Components; Table S1: Basic Performances of the KNN Sample; Table S2: Basic Performances of the Terfenol-D Sample; Table S3: The coil parameters; Table S4: Components and dimensions of structural materials. In the Supplementary Materials, we elaborate on KNN’s fabrication and composition, together with dimensions and intrinsic properties of KNN annular wafers, Terfenol-D and structural materials. Additional information including electromechanical performance, density, Curie temperature and sound velocity of the adopted materials, as well as component size specifications of the K-T-K transducer, is also provided.

## Abbreviations

The following abbreviations are used in this manuscript:

PZT	Lead Zirconate Titanate
UUVs	Unmanned Underwater Vehicles
THD	Total harmonic distortion
CW	Continuous-wave

## References

[1] Butler J.L., Butler A.L., Butler S.C. (1993). Hybrid magnetostrictive/piezoelectric Tonpilz transducer. J. Acoust. Soc. Am..

[2] Butler S.C., Tito F.A. (2000). A broadband hybrid magnetostrictive/piezoelectric transducer array. Proceedings of the OCEANS 2000 MTS/IEEE: Where Marine Science and Technology Meet, Providence, RI, USA, 11–14 September 2000.

[3] Mortensen A.P., Dapino M.J. (2005). Hybrid polymer matrix Terfenol-D composite/PMN-PT transducer in mechanical series configuration. Proc. SPIE.

[4] Chai Y., Mo X., Liu Y., Cui Z. (2006). A hybrid magnetostrictive-piezoelectric barrel-stave projector. Chin. J. Acoust..

[5] Tong H., Lan Y., Gu Z. (2010). Research on hybrid excited longitudinal vibration transducer. Chin. J. Appl. Acoust..

[6] Li Y., Xia D., Yu X. (2023). Magnetic energy losses and temperature control system for giant magnetostrictive transducer. Micromachines.

[7] Wei Y., Yang X., Chen Y., Zhang Z., Zheng H. (2022). Modeling of High-Power Tonpilz Terfenol-D Transducer Using Complex Material Parameters. Sensors.

[8] Liao Q., Guo L., Hong T., Wu J., Zhuo X., Li F. (2020). Full characterization for material constants of a promising KNN-based lead-free piezoelectric ceramic. Ceram. Int..

[9] Ma X., Yu X., Zhang H., Zhang M., Tang L., Li Q., Liu Z., Guo Y. (2026). Thermal stability and high-power performance of KNN-based lead-free piezoceramic lightweight ring transducer. Sens. Actuators A Phys..

[10] Liu D., Zhu L.F., Tang T., Li J.R., Wang L., Liu Y.X., Hao J., Wang S.D., Wang K. (2024). Textured potassium sodium niobate lead-free ceramics with high d_33_ and Q_m_ for meeting high-power applications. ACS Appl. Mater. Interfaces.

[11] Yang H., Tang L., Han S., Hua Y., Lin J., Qian J., Shen B., Jiang G., Zhai J. (2025). Ultra-stable thermal piezoelectricity up to 200 °C in high-performance KNN-based textured piezoceramics. Adv. Funct. Mater..

[12] Wang B., Wang J., Zhang H., Guo Y. (2025). Defect dipole-induced ultrahigh electrostrain in KNN-based piezoceramics for co-fired multilayer actuators with nickel electrodes. Ceram. Int..

[13] Cen Z., Cao F., Feng M., Li Z., Xu Z., Luo G., Luo N., Xie K., Li L., Wang X. (2023). Simultaneously improving piezoelectric strain and temperature stability of KNN-based ceramics via defect design. J. Eur. Ceram. Soc..

[14] Wu Y., Cheng Y., Guan S., Wang X., Shi W., Xu H., Lang R., Xing J., Zhu J., Chen Q. (2023). KNN-based lead-free piezoelectric ceramics with high Q_m_ and enhanced d_33_ via donor–acceptor codoping strategy. Inorg. Chem..

[15] Chen R., Shung K.K., Ma T., Zhou Q., Jiang L., Zhang T., Matsuoka T., Yamazaki M., Qian X., Lu G. (2019). Eco-friendly highly sensitive transducers based on a new KNN-NTK-FM lead-free piezoelectric ceramic for high-frequency biomedical ultrasonic imaging applications. IEEE Trans. Biomed. Eng..

[16] Quan Y., Xing J., Fei C., Tan Z., Sun Y., Cheng Y., Zhao T., Sun X., Zhao J., Zhang J. (2025). Graded KNN-based lead-free ultrasonic transducer for wide-temperature nondestructive testing imaging. ACS Appl. Mater. Interfaces.

[17] Xu L., Lin J., Yang Y., Zhao Z., Shi X., Ge G., Qian J., Shi C., Li G., Wang S. (2024). Ultrahigh thermal stability and piezoelectricity of lead-free KNN-based texture piezoceramics. Nat. Commun..

[18] Chen K., Ma J., Shi C., Wu W., Wu B. (2021). Enhanced temperature stability in high piezoelectric performance of (K,Na)NbO_3_-based lead-free ceramics through co-doped antimony and tantalum. J. Alloys Compd..

[19] Yang M., Yang X., Wei Z., Zhang Z., Chen Y. (2022). SPICE modeling of a high-power Terfenol-D transducer considering losses and magnetic flux leakage. IEEE Trans. Ultrason. Ferroelectr. Freq. Control.

[20] Li D., Li J. (2025). Research on active magnetic field compensation for high-efficiency Terfenol-D magnetic circuit in magnetostrictive transducers. J. Acoust. Soc. Am..

[21] (2002). Acoustics-Measurement of Underwater Sound Transducers.

[22] Butler J.L., Sherman C.H. (2016). Transducers and Arrays for Underwater Sound.

[23] Abdullah Z., Naz S., Raja M.A.Z., Zameer A. (2021). Design of wideband Tonpilz transducers for underwater SONAR applications with finite element model. Appl. Acoust..

[24] Ge X.H., Li J.B., Li D.P., Zhu H. (2026). Modal excitation and variable-band operation of piezoelectric Tonpilz transducers. J. Acoust. Soc. Am..

[25] Teng D., Zhu N. (2016). Research on the low frequency broadband piezoelectric-magnetostrictive hybrid transducer. Discret. Dyn. Nat. Soc..

