# Correction: Ma et al. A Lightweight, Low-Frequency, Broadband Underwater Acoustic Transducer with Ternary Symmetric Excitation: Integrating KNN and Terfenol-D for Enhanced Performance. *2026*, *26*, 3645

**DOI:** 10.3390/s26134200

**Published:** 2026-07-03

**Authors:** Xiongchao Ma, Zhenjun Liu, Shaobo Tang, Chenqi Shan, Qichao Li, Yiping Guo

**Affiliations:** 1 State Key Laboratory of Metal Matrix Composites, School of Materials Science and Engineering, Shanghai Jiao Tong University, Dong Chuan Road 800, Shanghai 200240, China; ma_xiongchao@163.com (X.M.); liqichao@sjtu.edu.cn (Q.L.); 2 Shanghai Marine Electronic Equipment Research Institute, Jindu Road 5200, Shanghai 201108, China; 13636393126@139.com (Z.L.); 13817918707@163.com (S.T.); s2338757836@foxmail.com (C.S.)

**Figure Legend**

In the original publication [1], there was a mistake in the legend for Figure 4. Displacement superposition of ternary symmetrical excitation. The sign of curve *X*_1_(t) is inverted, and the superposition of the superimposed curves contains errors. The correct Figure 4 appears below. The authors state that the scientific conclusions are unaffected. This correction was approved by the Academic Editor. The original publication has also been updated.

**Text Correction**

There were two errors in the original publication [1]. First, there exists a typographical error of a redundant “cc” in the paragraph.

A correction has been made to 2. Materials and Methods, 2.2. Dual-Resonance Low-Frequency Broadband Operating Mechanism, Paragraph 4:

If $f_{0}=\frac{1}{2\pi }\sqrt{k_{1}/m_{1}}$, under this condition, the following relation can be derived:

Second, subscripts and mathematical symbols are omitted in the section. The correct expression is *B* = *X_2_* rather than *B* = *X*—similarly, *X*_2_ = −cos(*wt*) instead of *X*_2_ = cos(*wt*). 

A correction has been made to 2. Materials and Methods, 2.2. Dual-Resonance Low-Frequency Broadband Operating Mechanism, Paragraph 8:

Herein, *A* = 2*X*_1_, *B* = *X*_2_, and *w* denotes the angular vibration frequency. Accordingly, this excitation scheme allows efficient amplitude superposition of the three-segment materials, with only a limited phase shift relative to the driving signal, as shown in Equations (6) and (7). Set *X*_1_ = sin(*wt*) and *X*_2_ = −cos(*wt*).

The authors state that the scientific conclusions are unaffected. This correction was approved by the Academic Editor. The original publication has also been updated.

## Figures and Tables

**Figure 4:**
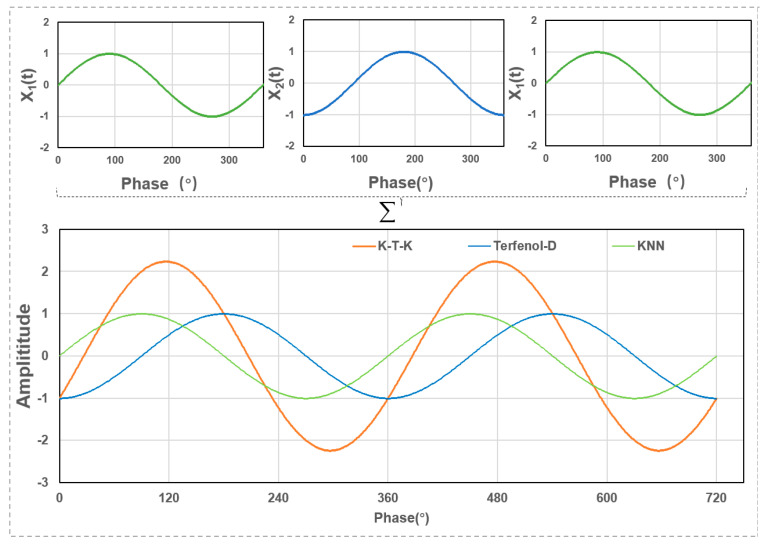
Displacement superposition of ternary symmetrical excitation.
