# Supplementary material for: A Lightweight, Low-Frequency, Broadband Underwater Acoustic Transducer with Ternary Symmetric Excitation: Integrating KNN and Terfenol-D for Enhanced Performance
Source: Sensors (Basel). 2026 Jun 7;26(12):3645. doi: 10.3390/s26123645 (PMC13306911; doi:10.3390/s26123645)
Supplement: Supplementary file 1 [file sensors-26-03645-s001.zip › sensors-4336477-supplementary.pdf]

Supplementary material

A Lightweight, Low-Frequency, Broadband Underwater Acoustic Transducer with Ternary Symmetric Excitation: Inte-grating KNN and Terfenol-D for Enhanced Performance

Xiongchao Ma[1,2], Zhenjun Liu[2], Shaobo Tang[2], Chenqi Shan[2], Qichao Li[1], and Yiping Guo[*1]

[1] State Key Laboratory of Metal Matrix Composites, School of Materials Science and Engineering, Shanghai Jiao Tong University, Dong Chuan Road 800, Shanghai 200240, China

[2] Shanghai Marine Electronic Equipment Research Institute, Jindu road 5200, Shanghai 201108, China

* Correspondence: ypguo@sjtu.edu.cn (Y. Guo)

1. *Composition and Preparation of KNN Materials, and Source of Terfenol-D*

The chemical composition of the KNN-based lead-free piezoelectric ceramic adopted in this work is $0.955(K_{0.48}Na_{0.52})(Nb_{0.99}Sb_{0.01})O_3$-$0.045(Bi_{0.5}Na_{0.5})ZrO_3$ doped with 0.5 mol% MnO_2, which is abbreviated as KNNS-BNZ-MnO_2. All ceramic samples were synthesized in our laboratory via a conventional solid-state reaction method. Post-annealing treatment in air was further employed to modulate lattice defects and optimize electrical properties of Mn-doped KNN-based lead-free piezoelectric ceramics. Raw materials including analytically pure K_2CO_3 (99.5%, Macklin), Na_2CO_3 (99.5%, Macklin) and Nb_2O_5 (99.9%, Macklin) were used as the matrix precursors, and MnO_2 served as the dopant. The starting powders were weighed according to the stoichiometric ratio and homogenized by wet ball milling. After drying, the mixed powders were calcined at 800–900 °C to complete the solid-phase synthesis of KNN-based powders. The calcined powders were re-milled for particle refinement, followed by granulation with binder addition and dry pressing to form green bodies. After binder burnout, the green bodies were sintered at 1100–1200 °C in air to obtain dense Mn-KNN ceramics. Subsequently, the sintered samples were annealed in air at 550 °C (denoted as T1-Mn-KNN) and 850 °C (denoted as T2-Mn-KNN), respectively. This post-annealing process regulated the recombination of oxygen vacancies, as well as the valence state and site occupation of Mn-related defects. Finally, the specimens were coated with silver electrodes and poled under a high electric field to fabricate high-performance piezoelectric ceramics with the composition of $0.955(K_{0.48}Na_{0.52})(Nb_{0.99}Sb_{0.01})$

O_3-0.045($Bi_{0.5}Na_{0.5}$)ZrO_3 doped with 0.5 mol% MnO_2, which is abbreviated as KNNS-BNZ-MnO_2.In addition, Terfenol-D was purchased from Suzhou Xunshi New Material Co., Ltd.

2. *Dimensions and Performance Parameters of Functional Materials*

The KNN annular sheet used in this study has an outer diameter of 25 mm, an inner diameter of 17 mm, and a thickness of 5 mm. The KNN driver rod is composed of 12 such annular sheets with a total length of 60 mm. The KTK transducer is equipped with two such driver rods at both ends. The Terfenol-D bar is 8 mm in diameter and 78 mm in length. The basic properties of the two materials are as follows:

Table S1 Basic Performances of the KNN Sample

Property	KNN sample
K_{33}	0.67
d_{33}(pC/N)	290
Relative permittivity (ε_{33}^{T})	2450
Longitudinal Sound Velocity (m/s)	4364
Density (g/cm^3)	4.2
Curie temperature (°C)	260
Mechanical Quality Factor Q_m	108
Young's modulus (Y_{33}^{E})(Gpa)	80
loss tangent tanδ	0.03
Prestress (Mpa)	10

Table S2. Basic Performances of the Terfenol-D Sample

Property	Terfenol-D
K_{33}	0.75
Saturation magnetostriction(λ_s ,ppm)	1500
Relative magnetic permeability	6
Longitudinal Sound Velocity (m/s)	1695
Density (g/cm^3)	9.25
Curie temperature （°C）	380
Mechanical Quality Factor Q_m	4.2
Young's modulus (Y^{H})(Gpa)	35
Magnetic loss tangent (tanδ_m)	0.2
Prestress (Mpa)	10

Table S3. The coil parameters are listed in Table 1.

Property	Value
Number of turns	1000
wire diameter (mm)	1
Inductance(mH)	10
Coil DC linear resistivity (Ω /m,20℃)	0.021
Breakdown voltages (V)	9200
Heat shock (℃)	200
Cut through temperature （℃）	2min 230℃
Manufacturer	Beijin Hangyuan gaoke co.Ltd
Structural configuration and dimensions of the coil bobbin	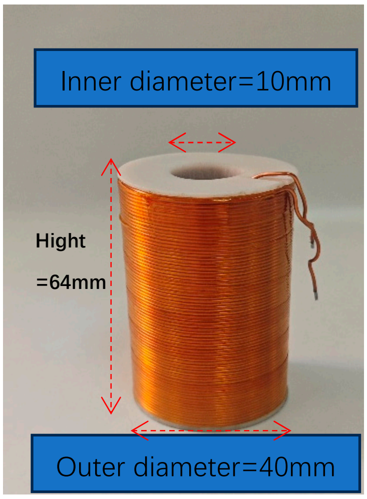

3. *Components and dimensions of structural materials*

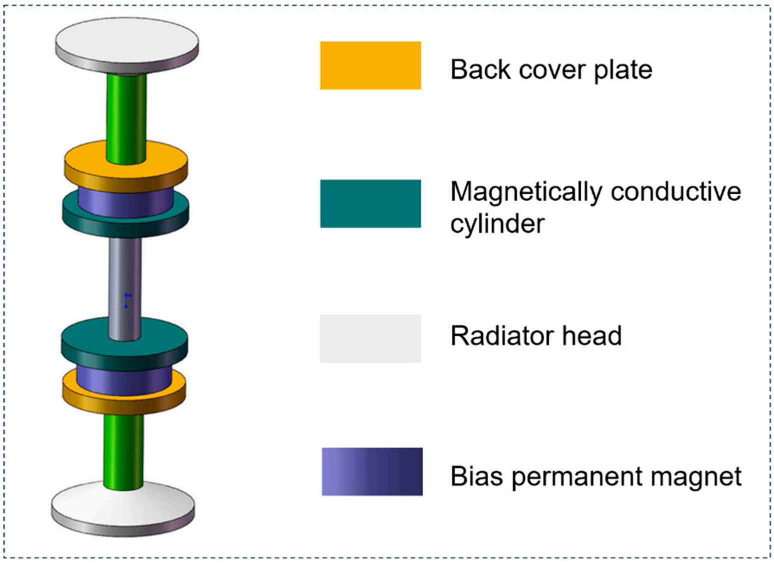

Figure. S1. Ternary symmetrically excited Components

Table S4. Components and dimensions of structural materials

Components	Materials and Type	Dimensions
Back cover plate	Copper<br>（HSn70-1）	Diameter: 50mm<br>Thickness:10mm
Magnetically conductive cylinder	Silicon steel<br>（30Q130）	Diameter: 50mm<br>Thickness:10mm
Radiator head	Aluminum<br>（7075）	Diameter: 80mm<br>Thickness:15mm
Bias permanent magnet	Neodymium-iron-boron magnet NdfeB42(The surface magnetic field strength is approximately 120 mT.)	Diameter: 50mm<br>Thickness:20mm
